# Monitoring Physical Activity Levels Using Twitter Data: Infodemiology Study

**DOI:** 10.2196/12394

**Published:** 2019-06-03

**Authors:** Sam Liu, Brian Chen, Alex Kuo

**Affiliations:** 1 School of Exercise Science, Physical and Health Education University of Victoria Victoria, BC Canada; 2 Department of Health Information Science University of Victoria Victoria, BC Canada

**Keywords:** physical activity, social media, internet, social media, Twitter messaging, population surveillance, public health

## Abstract

**Background:**

Social media technology such as Twitter allows users to share their thoughts, feelings, and opinions online. The growing body of social media data is becoming a central part of infodemiology research as these data can be combined with other public health datasets (eg, physical activity levels) to provide real-time monitoring of psychological and behavior outcomes that inform health behaviors. Currently, it is unclear whether Twitter data can be used to monitor physical activity levels.

**Objective:**

The aim of this study was to establish the feasibility of using Twitter data to monitor physical activity levels by assessing whether the frequency and sentiment of physical activity–related tweets were associated with physical activity levels across the United States.

**Methods:**

Tweets were collected from Twitter’s application programming interface (API) between January 10, 2017 and January 2, 2018. We used Twitter's *garden hose* method of collecting tweets, which provided a random sample of approximately 1% of all tweets with location metadata falling within the United States. Geotagged tweets were filtered. A list of physical activity–related hashtags was collected and used to further classify these geolocated tweets. Twitter data were merged with physical activity data collected as part of the Behavioral Risk Factor Surveillance System. Multiple linear regression models were fit to assess the relationship between physical activity–related tweets and physical activity levels by county while controlling for population and socioeconomic status measures.

**Results:**

During the study period, 442,959,789 unique tweets were collected, of which 64,005,336 (14.44%) were geotagged with latitude and longitude coordinates. Aggregated data were obtained for a total of 3138 counties in the United States. The mean county-level percentage of physically active individuals was 74.05% (SD 5.2) and 75.30% (SD 4.96) after adjusting for age. The model showed that the percentage of physical activity–related tweets was significantly associated with physical activity levels (beta=.11; SE 0.2; *P*<.001) and age-adjusted physical activity (beta=.10; SE 0.20; *P*<.001) on a county level while adjusting for both Gini index and education level. However, the overall explained variance of the model was low (*R*^2^=.11). The sentiment of the physical activity–related tweets was not a significant predictor of physical activity level and age-adjusted physical activity on a county level after including the Gini index and education level in the model (*P*>.05).

**Conclusions:**

Social media data may be a valuable tool for public health organizations to monitor physical activity levels, as it can overcome the time lag in the reporting of physical activity epidemiology data faced by traditional research methods (eg, surveys and observational studies). Consequently, this tool may have the potential to help public health organizations better mobilize and target physical activity interventions.

## Introduction

### Background

Physical inactivity is a modifiable risk factor for developing a widening variety of chronic conditions including cardiovascular diseases, hypertension, type 2 diabetes mellitus, colon cancers, osteoporosis, and depression [1-5]. Currently, many adults in the United States are physically inactive and do not meet the recommended amount of physical activity (150 min of moderate-intensity aerobic exercise per week) [6]. Furthermore, the prevalence of physical activity varies across geographic regions in the United States [7]. This lack of uniformity in the rate of physical activity in various geographic regions has become one of the top priorities for public health agencies—to collect population-level physical activity data. This epidemiology data can help identify groups and populations who are not engaged in regular physical activity and locations where these individuals live [8-10]. Local public health agencies can use this information to deploy appropriate resources to target health promotion efforts to improve physical activity levels in physically inactive regions. In fact, several studies have demonstrated the feasibility of using social media and internet-based interventions to promote physical activity on a population level [11-15]. Real-time epidemiology data of groups and locations of individuals who are not engaged in regular physical activity may further enhance public health agencies’ capabilities to personalize and target their interventions.

Existing methods of using population-based survey studies to monitor physical activity need to be improved. There are several limitations in current methods of collecting physical activity data [10,16]. First, reporting physical activity survey data in the United States involves up to 2 to 3 years of lag time, whereas surveys themselves can be time-consuming and resource-intensive to conduct. Sparsity of data can be challenging for many surveys, as response rates may not vary uniformly by location, demographics, or population. Therefore, innovative research approaches are needed to supplement and improve the current state of physical activity monitoring.

Social media use has grown rapidly in the last decade, [17] and researchers have been examining ways to use *social data* to better understand and monitor public health problems in *real-time* [16,18]. This growing area of research has been called *infodemiology* or *infoveillance* studies [19,20]. Social media technology, such as Twitter, allows users to communicate with each other by sharing short messages. Users can share their thoughts, feelings, and opinions on these social media platforms and, as a result, social media data may be used to provide real-time monitoring of behavioral outcomes that inform health behaviors [21]. A unique aspect of social media data from Twitter is that the posts are public and geotagged and thus, all internet users, including health researchers, can readily access these data. In addition, unique to Twitter is the use of hashtags (*#*) that allow a user to highlight and allow other users to follow relevant topics of interest. Given their high level of use, these sites collect an enormous amount of data (eg, over 500 million tweets per day on Twitter) [21].

Recent infodemiology studies have reported that data from social media technologies can be combined with other biomedical datasets to help predict health outcomes [16,22-25]. The main approaches to analyze unstructured text data from Twitter include frequency of keyword occurrence (analysis of information prevalence and information occurrence ratio) and the sentiment of the tweets [10,19]. These approaches are not mutually exclusive and thus can be used together for monitoring physical activity. Information prevalence and information occurrence ratios measure the absolute or relative *frequency* of occurrences of a certain keyword. The amount of social data is constantly increasing; thus, normalized indicators (eg, information occurrence ratio) may be more meaningful than absolute figures on information prevalence [19]. Finally, sentiment analysis can determine whether an individual’s attitude or perception toward a topic is positive, negative, or neutral. By applying these methods, researchers have shown that social data can be used to identify symptoms associated with psychological distress, anxiety, and depression [22] and identify infectious disease outbreaks, such as influenza transmission [26,27] and HIV outbreaks [24]. Previous studies have also reported that the frequency of physical activity–related tweets and the sentiment of the tweets are related to obesity rates [28], social disparity, and wellness indicators in US Metropolitan Statistical Areas (MSAs) [29,30]. Currently, it remains unclear whether these methods of analyzing physical activity–related tweets can be applied to monitor the physical activity level on a county level across the United States while controlling for socioeconomic inequality and education level.

### Objectives

The aim of this study was to establish the feasibility of using Twitter data to monitor physical activity levels by assessing whether the frequency and sentiment of physical activity–related tweets were associated with physical activity levels in various counties across the United States.

## Methods

### Overview

Tweets (n=442,959,789) were collected from January 10, 2017, to January 2, 2018, using Twitter’s application programming interface (API). The captured tweets represent an estimated 1% random selection of all tweets posted in a selected time frame. Only *geolocated* tweets with coordinates or within the bounding box defined by –162.354635, 18.756125, –53.755999, 73.893030 were retained for analysis. Additional processing was applied to filter out tweets with coordinates not originating from the United States, leaving a final sample of 64,005,336 tweets. To categorize tweets on a county level, a reverse-geocoding pipeline using cartographic boundary shapefiles from the US Census Bureau was created to assign a Federal Information Processing Standard code for each tweet.

### Classifying Physical Activity–Related Tweets

A list of physical activity–related hashtags was compiled (see Multimedia Appendix 1) to identify tweets that might be related to exercise or physical activity. The hashtags were compiled using a combination of the most popular physical activity–related hashtags and the guidelines for exercise testing published by the American College of Sports Medicine (ACSM) [31]. The ACSM guideline was used because it provided an extensive list of physical activity–related keywords that were well established, and this method has been used in previous research [28,31]. A tweet was classified as a physical activity–related tweet if it contained one or more physical activity–related keyword in the tweet’s hashtags. Although previous studies have relied on dictionaries of exercise-related keywords (eg, from the Compendium of Physical Activities and ACSM guidelines for exercise testing) to classify tweets, using hashtags presents a couple of important advantages: they can be parsed as distinct entities from tweets and can represent more specific multi-word phrases [28,29]. As such, there is less risk of ambiguity with hashtags than with a dictionary or list of keywords (eg, *walk* and *surf* may have multiple meanings outside of physical activity, whereas *#30daysoffitness* is unlikely to). Previous research has also discussed the difficulty of this classification task, either electing to improve precision by imposing additional rules (eg, requiring additional context for commonly ambiguous terms such as *running*) on top of the basic dictionary word check list or choosing not to apply any additional filtering to avoid introducing additional biases into the sampling methodology [29]. Although using hashtags does not rectify the issue of curation bias, it does allow for far more specific matching against text than regular words, while also maintaining the simplicity of a simple list of items. This inherently trades off increased precision at the loss of recall or sensitivity but ensures that fewer unrelated tweets are passed to the sentiment analysis pipeline.

### Sentiment Analysis

Using sentiment analysis techniques to study microblogging services such as Twitter is a rich and active area of study. Sentiment analysis assigns text documents polarities, labels such as *positive*, *negative*, and *neutral* that describe the writer’s attitude as written. When applied to a topic, sentiment analysis may be used to predict or infer these attitudes based primarily on a collection of documents. This study utilized a sentiment analysis model created by Baziotis, Pelekis, and Doulkeridis for the 2017 International Workshop on Semantic Evaluation (SemEval) [32,33]. This model ranked first in Subtask A of Task 4 (*Sentiment Analysis in Twitter*) at SemEval 2017 and employs a bidirectional long short-term memory neural network with an attention mechanism [34].

### Physical Activity Dataset

Physical activity levels and age-adjusted physical activity levels were extracted from the Behavioral Risk Factor Surveillance System (BRFSS) surveys, which provides county-level data of physical activity levels from the year 2014. The BRFSS is administered by the Centers for Disease Control and Prevention. As part of the survey, participants were asked to self-report leisure-time physical activity (eg, during the past month, other than your regular job, did you participate in any physical activities or exercises such as running, calisthenics, golf, gardening, or walking for exercise?). Self-reported leisure-time physical inactivity was ascertained from answers of *no* to the questions. The BRFSS physical activity data were collected for adults aged 18 years and older, thus BRFSS also reported age-adjusted physical activity data based on the US standard population. Socioeconomic status measures, such as the Gini index, were collected from the American Community Survey.

### Statistical Analysis

The frequency of physical activity tweets was tallied from each county and merged with physical activity levels from the BRFSS data, Gini index data, and percent of the county that received college education. The Gini index provides a standardized estimate of income inequality that may be used for comparisons between counties. Including the Gini index and education level is pertinent in the context of physical activity; these variables have been associated with levels of physical activity [35].

A bivariate Spearman correlation was used to determine the association between the number of physical activity–related tweets (including the number of positive, negative, and neutral tweets), the Gini index, education, and physical activity data. Multiple linear regression models were then applied to find the level of association between the proportion of physical activity–related tweets, sentiment of the tweets (ratio of positive to negative physical activity–related tweets), and physical activity data while controlling for the Gini index and education level on a county level. The relative performance of these models was compared. All analyses were performed using IBM SPSS 24.0 (IBM Corporation).

## Results

**Figure 1:**
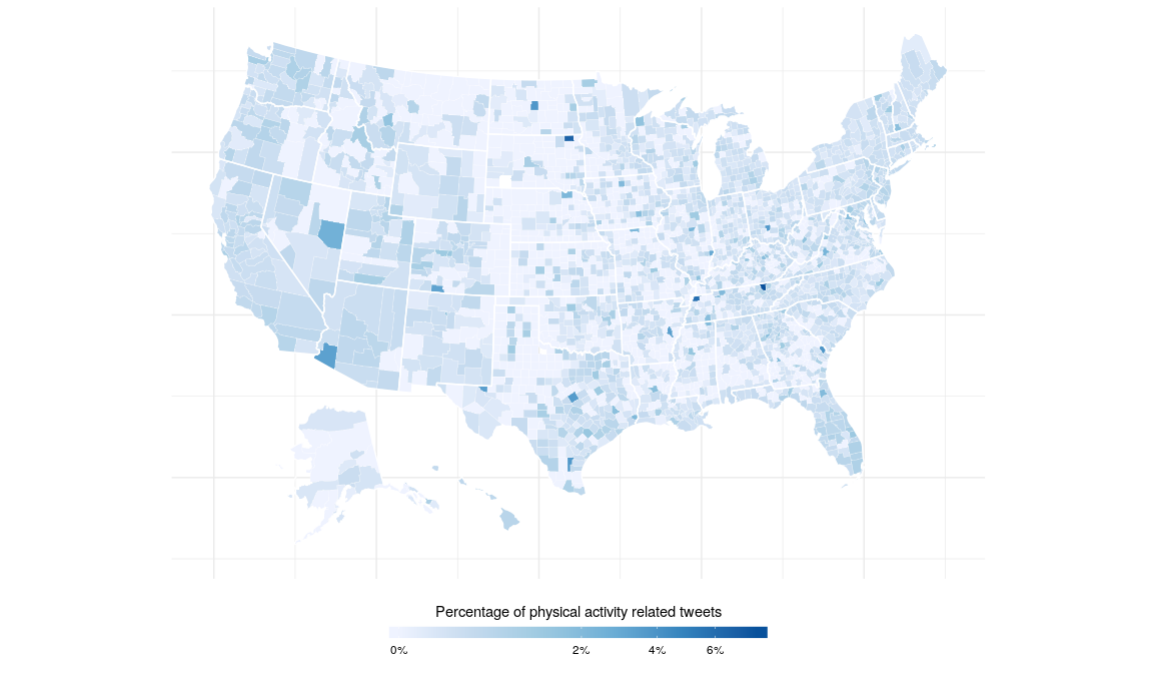
Map of physical activity levels in the United States.

Of the 442,959,789 unique tweets collected, 64,005,336 (14.44%) were geotagged. Of these, 234,678 (0.37%) were identified to be physical activity–related based on their hashtags. Los Angeles County (n=20,589; 8.77%), New York County (n=12,601; 5.37%), Miami–Dade County (n=7055; 3.01%), Harris County (n=6148; 2.62%), and Cook County (n=5738; 2.45%) were the 5 counties that sent the most geotagged physical activity–related tweets (Figure 1).

**Figure 2:**
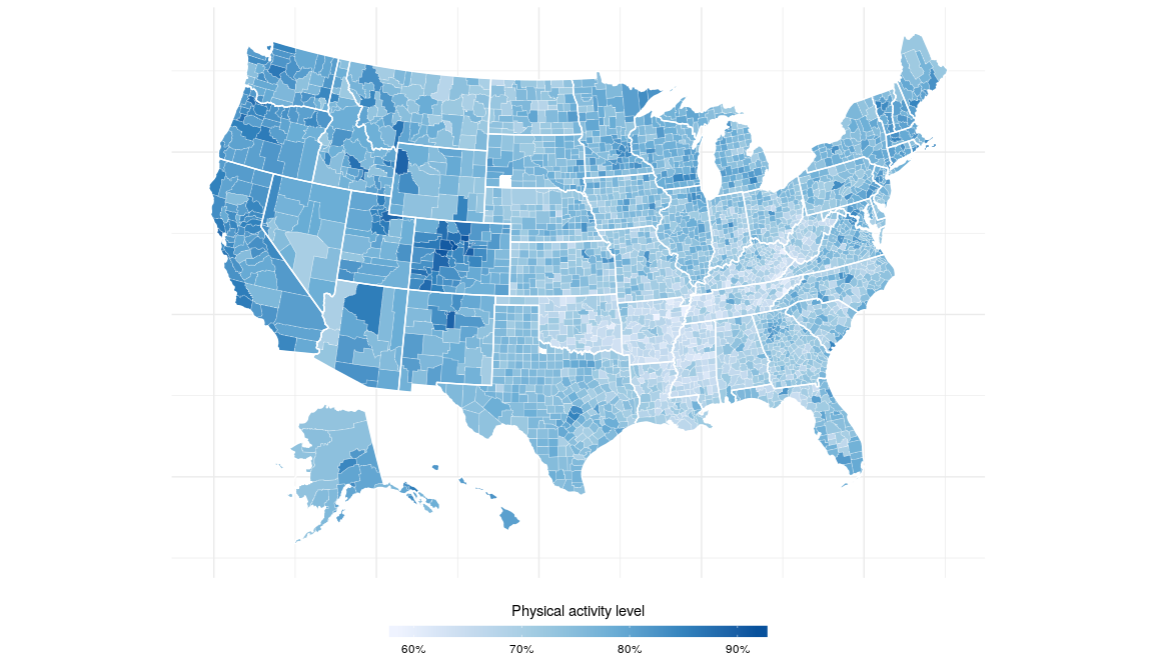
Map of physical activity–related geolocated tweets in the United States.

**Figure 3:**
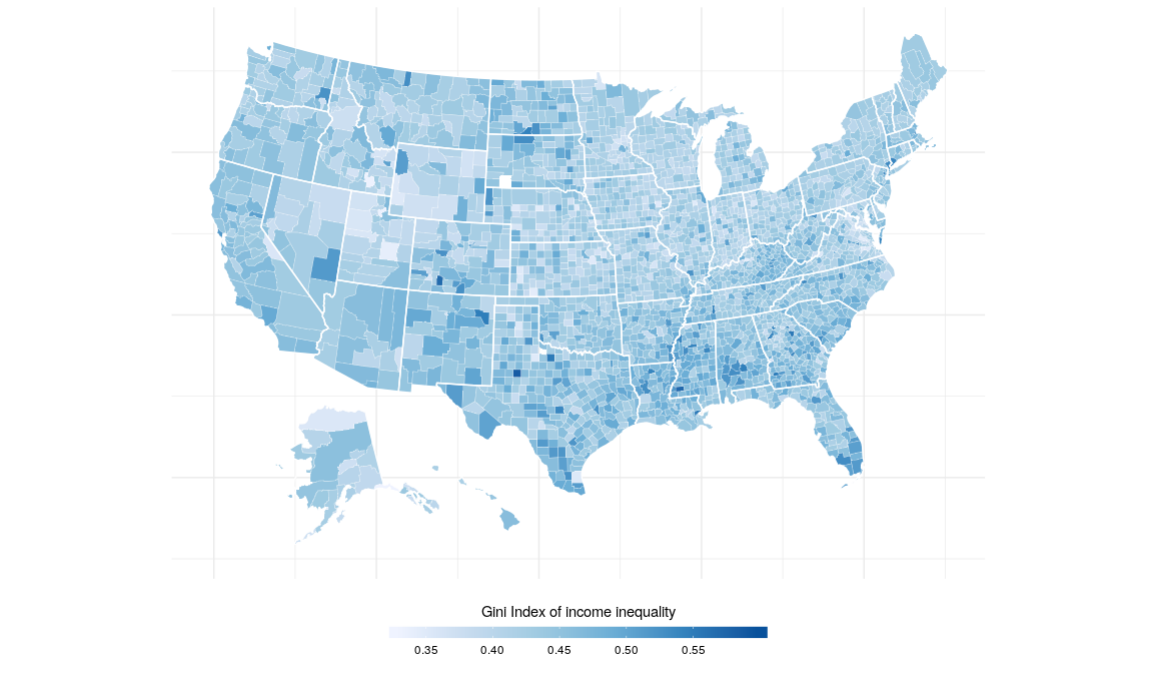
Map of Gini index across the United States.

**Figure 4:**
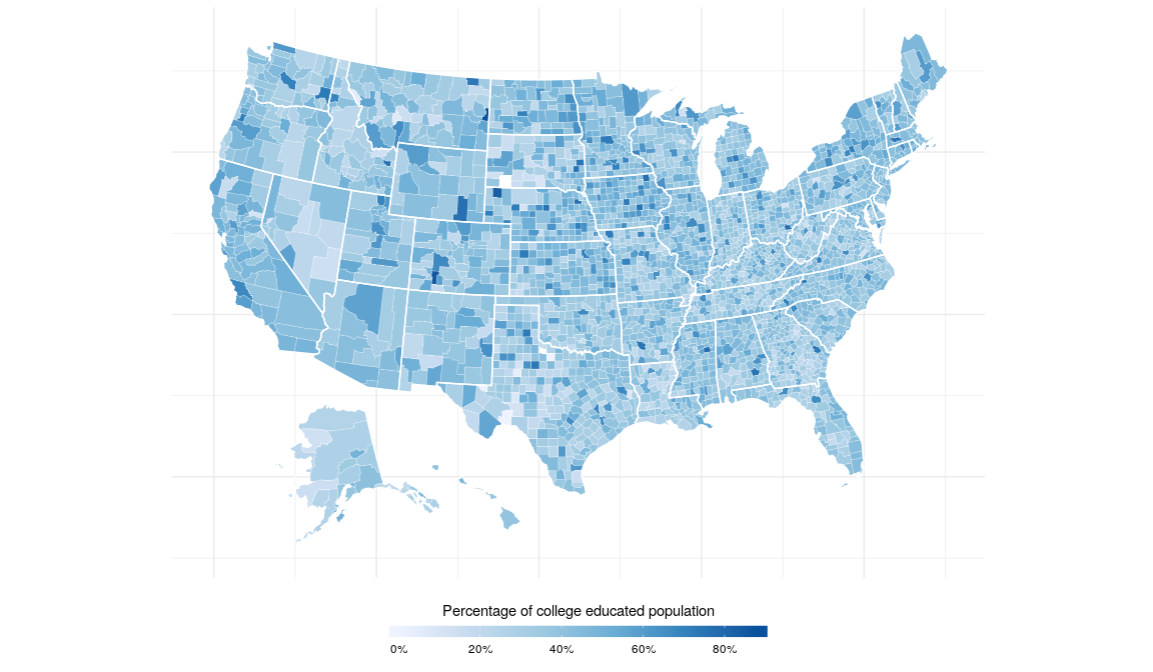
Map of education level across the United States.

Aggregated data were obtained for a total of 3138 out of 3146 counties or county equivalents. The counties omitted for analysis lacked correlated Twitter data, physical activity data, or data on the socioeconomic indicators. The mean county-level percentage of individuals that are physically active was 74.05% (SD 5.2) and 75.30% (SD 4.96) after adjusting for age (Figure 2). Maps of the Gini index and education levels are displayed in Figures 3 and 4.

Our sentiment analysis showed that 7.31% (n=17,155) of the physical activity–related tweets identified were positive, 42.67% (n=100,137) were negative, and 50.02% (n=117,386) were neutral. The mean ratio between positive and negative was 0.20 (SD 0.336). Textbox 1 shows example tweets. On the basis of the correlation analysis, county-level physical activity and age-adjusted physical activity level showed a significant positive weak-to-moderate correlation with the percentage of physical activity–related tweets and the sentiment of physical activity–related tweets (Table 1).

**Example tweets classified with positive, negative, and neutral sentiment.**

Classified positive:

- “Growth physically, mentally, spiritually, financially. That will be my 2017....#instafit…”
- “I think between us we've lost a whole person! #fitgoals…”
- “Today's reminder: it's about practice, not perfection! #yogajournal…”

Classified negative:

- “I hate Tuesdays. Extra. #cardio”
- “Shoutout to #crossfit ... More like curbstomp am I right guys?! @ Portland, Oregon”
- “Back to the grind… #cardio”

Classified neutral:

- “Where’s the #belly I see it! #handful but in a good way #fitness #goals continue…”
- “Day 2 of my 2 a day workouts down #gymlife #planetfitness…”
- “Clips of my leg day #squatsanddeads #bodybuilding #powerlifting #strongman #olympiclicting #fit…”

**Table 1:** Summary of county-level physical activity, activity-related tweets, and the Gini index.

Variable	Physically active, %	Physically active, % (age adjusted)	Gini index	Education	Physical activity tweets	The ratio between positive and negative physical activity tweets
Physically active, %	1	0.99^a^	–0.16^a^	0.26^a^	0.38^a^	0.13^a^
Physically active, % (age adjusted)	—^b^	1	–1.77^a^	0.24^a^	0.34^a^	0.10^a^
Gini index	—	—	1	0.04^c^	0.05^c^	0.09^a^
Education	—	—	—	1	0.22^a^	0.16^a^
Physical activity tweets	—	—	—	—	1	0.20^a^
Positive / negative physical activity tweets ratio	—	—	—	—	—	1

^a^*P*<.001.

^b^Not applicable.

^c^*P*<.02.

The regression models showed that the percentage of physical activity–related tweets was significantly associated with the physical activity level (Table 2) and age-adjusted physical activity on a county level (Table 3) while adjusting for both the Gini index and education level. However, the sentiment of the physical activity–related tweets was not a significant predictor of the physical activity level and age-adjusted physical activity on a county level after including the Gini index and education level in the model. The best-fit model for predicting county-level physical activity incorporated the percentage of physical activity–related tweets, the Gini index, and the prevalence of college education. However, the overall explained variance of the model was low (*R*^2^=.11) Similarly, the best-fit model for predicting county-level physical activity (*R*^2^=.09) after adjusting for age used the percentage of physical activity–related tweets, the Gini index, and the education level.

**Table 2:** Regression analysis for physical activity–related tweets and county-level physical activity level.

Variables		beta	SE	*P* value
**Model 1^a^**				
	Gini index	−0.16	2.54	<.001
	Education	0.26	.01	<.001
	Percent of physical activity–related tweets	0.11	.20	<.001
**Model 2^b^**				
	Gini index	−0.12	3.78	<.001
	Education	2.95	.01	<.001
	Sentiment of physical activity–related tweets (positive/negative ratio)	−0.01	.37	.56
**Model 3^c^**				
	Gini index	−0.12	3.79	<.001
	Education	0.30	0.01	<.001
	Percent of physical activity–related tweets	0.05	.37	.02
	Sentiment of physical activity–related tweets (positive/negative ratio)	−0.01	.24	.53

^a^*F*_3,3137_=116.30; *P*<.001; *R*^2^=.11.

^b^*F*_3,1704_=55.99; *P*<.001; *R*^2^=.09.

^c^*F*_3,1704_=43.517; *P*<.001; *R*^2^=.09.

**Table 3:** Regression analysis for physical activity–related tweets and age-adjusted county-level physical activity level.

Variables		beta	SE	*P* value
**Model 1^a^**				
	Gini index	−0.18	2.44	<.001
	Education	0.23	.01	<.001
	Percent of physical activity–related tweets	0.10	0.20	<.001
**Model 2^b^**				
	Gini index	−0.13	3.63	<.001
	Education	0.25	.01	<.001
	Sentiment of physical activity–related tweets (positive/negative ratio)	−0.02	.35	.44
**Model 3^c^**				
	Gini index	−0.13	3.64	<.001
	Education	0.26	.01	<.001
	Percent of physical activity–related tweets	0.05	.23	.03
	Sentiment of physical activity–related tweets (positive/negative ratio)	−0.02	.35	.41

^a^*F*_3,3137_=102.93; *P*<.001; *R*^2^=.10.

^b^*F*_3,1704_=43.09; *P*<.001; *R*^2^=.07.

^c^*F*_3,1704_=33.52; *P*<.001; *R*^2^=.07.

## Discussion

### Principal Findings

This study evaluated the feasibility of using Twitter data to monitor physical activity levels by assessing whether geotagged conversations about physical activity behaviors can be extracted from Twitter and whether physical activity–related tweets could be used to monitor physical activity levels. Results suggest that it is feasible to extract physical activity–related geotagged conversations from Twitter. Furthermore, the results suggest that there was a significant association between physical activity–related tweets and physical activity levels while accounting for the Gini index of income inequality, population, and education on a county level across the United States. However, the overall association between physical activity–related tweets and physical activity levels on a county level was weak.

### Research Implications

Exploring the relationship between physical activity–related tweets and physical activity levels on a county level has several important research implications. First, these findings support the continued research in using nontraditional data sources, such as social media data, to monitor physical activity–related behaviors. Second, our results demonstrated a potential application for using social media data as a complementary tool to aid in both historical and real-time tracking of population-level physical activity. A strength of this study is controlling for related demographic factors such as income inequality and education in various geographic locations in our model. Finally, physical activity researchers can build upon the methods used in this study to find new methods of using social media data to monitor physical activity outcomes. Physical activity researchers can leverage these social media analysis techniques to build models that can predict physical activity levels in real-time. The analysis methods used in this study could in the future aid public health agencies in identifying particular physical activity–related trends or geographical areas of concern on which to focus their health and wellness initiatives.

Findings from this study validate and extend previously published work that the content of the tweets can be potentially used to monitor and predict behavior and health outcomes [10,16,22]. It is worth noting that even though we did not show a significant association between the sentiment of physical activity–related tweets and age-adjusted physical activity on a county level, previous studies have shown that the sentiment of the tweets can be used to predict health outcomes. Specifically, a previous study reported that positive sentiment tweets were moderately correlated with lower obesity rates in 190 US MSAs [28]. These findings suggest that sentiment analysis may not be an appropriate estimator of physical activity level on a county level but may still be an appropriate estimator in other health-related outcomes on an MSA level.

Although infoveillance or infodemiology studies such as this are important to epidemiology to avoid *ecological fallacies* [19], it is critical for future research to examine the relationship between social media data and physical activity level on an individual level. Studying the prevalence of physical activity is a complex and nuanced topic, one that may be strongly influenced by an individual’s surrounding environment. We were able to obtain improved model performance through the inclusion of per county Gini index data on income inequality and education level. However, future studies will need to investigate whether other known metrics or indicators (eg, the built environment) can be incorporated into Twitter data to create models with improved accuracy in predicting physical activity.

### Limitations

There are several limitations in the study. There was a lag time and time frame disparity between the Twitter data and physical activity data. The most recent county-level physical activity data, collected as part of its annual BRFSS surveys, was from 2014. In addition, there exist inherent biases that must be noted for any sampling of geotagged Twitter data. Studies on demographics on the platform have found a skew toward a younger, wealthier demographic in general [21], as well as increased representation from minority groups and urban populations when looking at geotagged tweets in particular [30]. This means that the observed relationship between physical activity–related tweets and physical activity needs to be interpreted with caution as certain demographic or regional groups may be predisposed toward a certain physical activity level. Nevertheless, this study was a feasibility study primarily designed to evaluate whether social media conversations that suggest physical activity–related behaviors could be extracted and used to monitor physical activity at a population level. Second, we only used one source of social media data (Twitter), thus limiting the generalizability of our findings. Using Twitter API’s *garden hose* approach with geographic filtering also limits data collection to less than a 1% random sample of all tweets posted in any given time frame. Future studies will need to explore whether model accuracy can be improved using multiple data sources (eg, Twitter, Facebook, and Instagram) to exploit user overlap between certain social media platforms as well as over a longer data collection period. Finally, studies of this nature rely heavily on the accuracy of the classifier for labeling physical activity–related tweets and conducting sentiment analysis. In particular, the finer-grained filtering offered by sentiment analysis did not appear to offer a notable improvement in fit or classification accuracy. It should be noted that this failure of complex or synthesized features to improve model quality has been observed earlier [28]. Subsequent studies may supplant our list-of-hashtags classifier with machine-learning classification approaches to potentially discover keywords, text structure, or other features that may be used to boost both precision and recall, as well as attempt using state-of-the-art sentiment analysis techniques to construct and train custom classifiers that are a better fit for this specific subset of Twitter data.

Moving forward, there are still other possible features to extract from Twitter data that may be tested for association with levels of physical activity. Although this study focused exclusively on filtering Twitter data by keywords and conducting sentiment analysis, there may be other natural language processing techniques that could be applied to the dataset [16,36]. Future research could investigate training predictive models on a larger, longitudinal dataset of both tweets and physical activity data. If successful, such models could be leveraged to effectively predict levels of physical activity and inactivity using social media data.

### Conclusions

This study evaluated the feasibility of using social media data to monitor physical activity levels on a county-by-county basis. Results from this study suggest that it is feasible to identify geotagged physical activity–related conversations from Twitter data and link them to population-based physical activity outcome data for analyses. We found that the conversation from tweets was weakly associated with county-level physical activity levels in the United States. Future research can build on the methods used in this study to further refine the models that use real-time social media data to monitor physical activity levels.

## Appendix

Multimedia Appendix 1 Hashtags used to identify tweets related to physical activity.

## Abbreviations

ACSM: American College of Sports Medicine
API: application programming interface
BRFSS: Behavioral Risk Factor Surveillance System
MSA: Metropolitan Statistical Area
SemEval: Semantic Evaluation
