# Supplementary material for: Monitoring Physical Activity Levels Using Twitter Data: Infodemiology Study
Source: J Med Internet Res. 2019 Jun 3;21(6):e12394. doi: 10.2196/12394 (PMC6682305; doi:10.2196/12394)
Supplement: Multimedia Appendix 1 [file jmir_v21i6e12394_app1.docx]

**APPENDIX 1**

**List of hashtags used for filtering physical activity-related tweets**

| **Hashtag** |
| --- |
| #30daysoffitness |
| #abs |
| #active |
| #aesthetic |
| #beastmode |
| #body |
| #bodybuilding |
| #bodytransformation |
| #cardio |
| #coaching |
| #crossfit |
| #exercise |
| #fit |
| #fitfam |
| #fitgirl |
| #fitlife |
| #fitness |
| #fitnessaddict |
| #fitnessprofessional |
| #fitpro |
| #fitspiration |
| #fitspo |
| #getfit |
| #girlsthatlift |
| #gym |
| #gymjunky |
| #gymlife |
| #gympost |
| #instafit |
| #journeystartshere |
| #juijitsu |
| #kickboxing |
| #lift |
| #mastertrainer |
| #muscle |
| #myfitnessjourney |
| #personaltraining |
| #physicalactivity |
| #protein |
| #ptbusiness |
| #run |
| #running |
| #selfdefense |
| #shredded |
| #sport |
| #strength |
| #toning |
| #training |
| #weightloss |
| #weightlossjourney |
| #weightlossmotivation |
| #weightlosstransformation |
| #workout |
| #yoga |
